# Leptin receptor neurons in the ventral premammillary nucleus modulate emotion-induced insomnia

**DOI:** 10.1038/s41421-024-00676-x

**Published:** 2024-06-04

**Authors:** Xiang-shan Yuan, Zhe Xiang, Jian-bo Jiang, Fang Yuan, Mu-tian Zhang, Kai-ying Zhang, Zhao-yi Chen, Wei-min Qu, Wen-sheng Li, Zhi-li Huang

**Affiliations:** 1 Department of Anatomy and Histoembryology, and Department of Pharmacology, School of Basic Medical Sciences; State Key Laboratory of Medical Neurobiology, Institutes of Brain Science and Collaborative Innovation Center for Brain Science; Department of Anesthesiology, Zhongshan Hospital, Fudan University, Shanghai, China; 2 Department of Neurobiology, Hebei Medical University, Shijiazhuang, Hebei China

**Keywords:** Circadian rhythms, Hormone receptors

Dear Editor,

Insomnia, a primary sign of sleep disorders, involves problems with sleep duration and onset. Exposure to intense emotional stimuli before bedtime has been shown to increase sympathetic activity, reduce restorative sleep, and heighten nighttime vigilance in humans^1^. Emotional triggers, such as predators or rewards, can also induce hyperarousal in animals^2,3^, indicating that emotional stimuli can lead to insomnia. External environmental stimuli, particularly pheromones, serve as significant triggers for emotional changes in mice, resulting in avoidance, aggressive behaviors, heightened attentiveness, and prolonged arousal time^4–6^. It is widely known that pheromones are relayed through specific neural pathways, including the vomeronasal organ, accessory olfactory bulb, medial amygdala, and bed nucleus of stria terminalis, and integrated into the ventral premammillary nucleus (PMv)^7^. Recent studies have verified that pheromones induce a remarkable increase in the activity of PMv neurons and subsequently lead to aggressive or reproductive behaviors^4,8,9^. These findings suggest that the PMv is crucial in integrating pheromone inputs and regulating aggressive or reproductive behaviors depending on alertness or wakefulness. However, it’s unclear whether the PMv regulates sleep–wake behavior and contributes to insomnia induced by emotional stimuli.

To invoke emotional triggers, we chose urine as mimic predator or reward signals, and assessed emotional features through conditioned place preference (CPP) including the value, intensity, and persistence (Supplementary Fig. S1a)^10,11^. Conspecific male or female mouse urine elicited preference, while male rat urine or TMT (a component of fox odor) induced avoidance (Supplementary Fig. S1b–d). Moreover, the preference/avoidance behavior gradually diminished as male mouse urine/TMT was diluted (Supplementary Fig. S1e–g). To evaluate the persistence of emotion, mice underwent CPP test without urine stimuli again after exposure, and still exhibited preference/avoidance behavior in the absence of male mouse urine/TMT (Supplementary Fig. S1h–l). These results indicate that urine/TMT, as sensory stimuli, effectively triggers emotional changes.

To determine the impact of emotional stimuli on sleep–wake behavior in mice, we monitored sleep–wake behavior in sexually inexperienced adult mice following exposure to control stimulus (saline) and various emotional stimuli (urine or TMT) (Fig. 1a). All emotional stimuli significantly delayed non-rapid eye-movement (NREM) sleep onset compared to the control group (Fig. 1b). Mice exposed to male mouse urine exhibited increased wakefulness and decreased in rapid eye movement (REM) and NREM sleep for 2 h compared to the control group (Fig. 1c and Supplementary Fig. S2). Similar effects were observed with other stimuli (female mouse urine, male rat urine, TMT) (Fig. 1c and Supplementary Fig. S2). These results confirm that emotional stimulation elicits insomnia-like behavior.

**Fig. 1:**
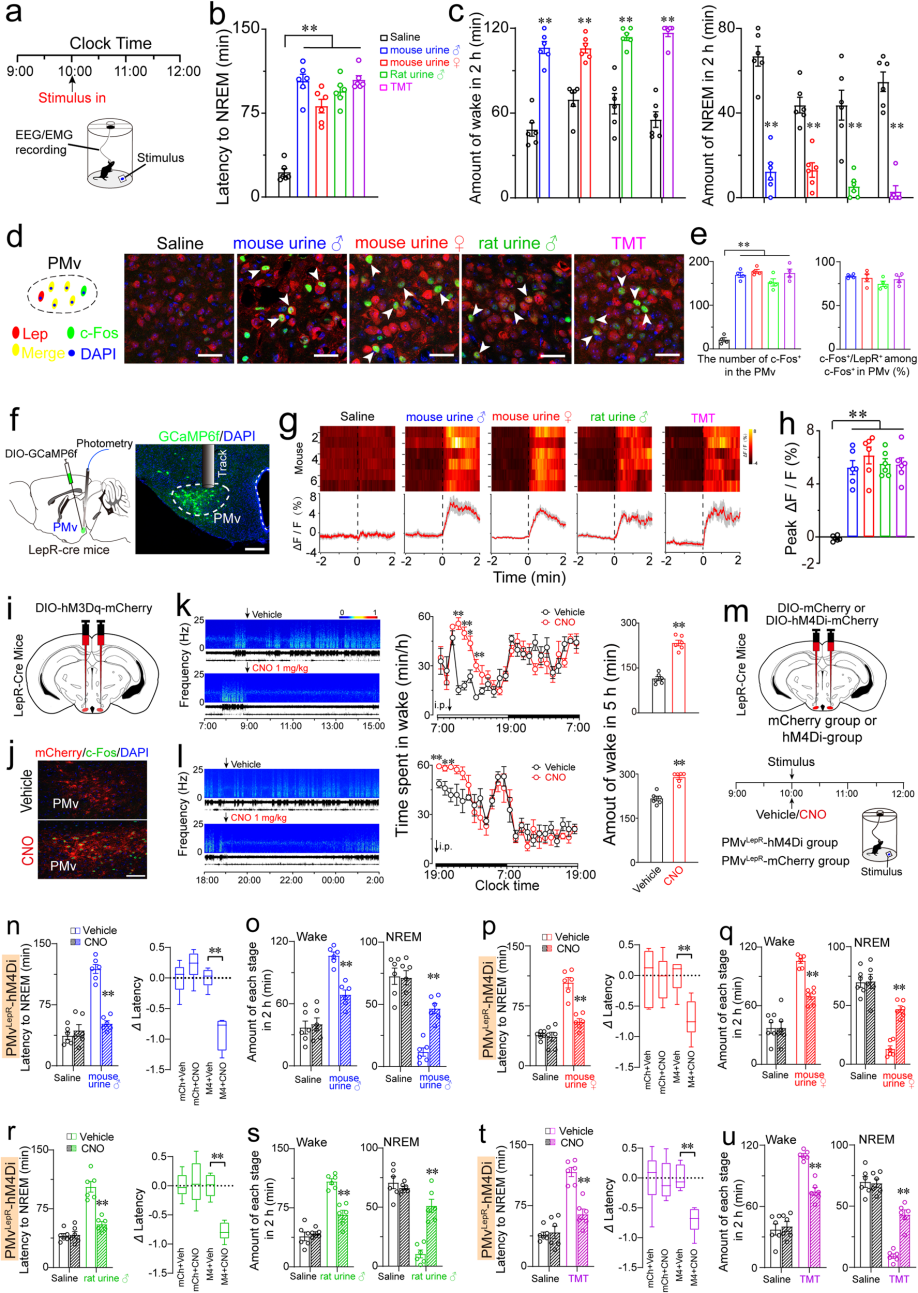
Inhibition of PMv^LepR^ neurons rescues emotion-induced insomnia-like behavior. **a** Schematic showing experimental protocol for EEG/EMG recording and emotional stimuli. **b** The latency of NREM sleep onset following male mouse urine (blue), female mouse urine (red), male rat urine (green), TMT (magenta) and saline (black) (*n* = 6, one-way ANOVA). To maintain consistency, identical color coding for each group is applied throughout all subsequent figure panels. **c** Total amount of wakefulness (left), NREM sleep (right) during 2 h (10:00–12:00) following various stimuli (*n* = 6, paired *t*-test). **d** Representative images showing co-localization (arrowheads) of c-Fos with LepR signals in the PMv following various stimuli. Scale bars, 50 µm. **e** Quantification of the number of c-Fos^+^ cells in the PMv for each treatment (left, *n* = 4, one-way AVONA) and the percentage of c-Fos^+^/LepR^+^ cells in the c-Fos^+^ population in the PMv for each treatment (right). **f** Schematic of fiber-photometry recordings and an image showing GCaMP6f (green) expression in PMv^LepR^ neurons of LepR-Cre mouse. Scale bar, 200 µm. **g** Heatmap (upper) of average $\Delta$*F*/*F* signal when animals sniffing different stimuli and saline, and average calcium transients (lower) when animals sniffing different stimuli and saline (*n* = 6, values of average $\Delta$*F*/*F* signal were averages of 3 trials for each animal). **h** Quantification of stimuli-induced peak $\Delta$*F*/*F* (%) for exposed animals (*n* = 6, one-way AVONA). **i** Schematic of viral strategy to chemogenetically activate PMv^LepR^ neurons. **j** Representative image showing that CNO induced c-Fos/hM3Dq-expressing neurons of the PMv. Scale bar, 100 μm. **k**, **l** Examples of relative EEG power, time course changes in wakefulness, amount of wakefulness during the 5 h following vehicle or CNO injections at light (**k**) and dark period (**l**) (*n* = 6, two-way repeated measures ANOVA, paired *t*-test). **m** Schematic of virus strategy in the PMv of LepR-Cre mice, with mCherry or hM4Di virus and the experimental timeline for stimuli or CNO treatment. **n**, **p**, **r**, **t** The latency of NREM sleep following vehicle or CNO in PMv^LepR^-hM4Di mice, and $\Delta$ Latency to NREM sleep of PMv^LepR^-mCherry and PMv^LepR^-hM4Di mice after male mouse urine exposure (**n**), female mouse urine exposure (**p**), male rat urine exposure (**r**), TMT exposure (**t**) or saline exposure (*n* = 6, ANOVA for factorial design). **o**, **q**, **s**, **u** Total amount of wakefulness and NREM sleep during 2 h (10:00–12:00) following vehicle or CNO in PMv^LepR^-hM4Di after male mouse urine exposure (**o**), female mouse urine exposure (**q**), male rat urine exposure (**s**), TMT exposure (**u**), or saline exposure (*n* = 6, paired *t*-test). Data represent mean ± sem, *P < 0.05, **P < 0.01.

To identify brain regions exhibiting acute activity in response to emotional stimuli, we examined the expression of c-Fos in the global brain after emotional stimuli. We found a significant increase in c-Fos^+^ cells in various brain regions, including the PMv, medial preoptic nucleus, medial amygdala, and locus coeruleus. Notably, the expression of c-Fos in the PMv was the highest increment among all nuclei, after different stimuli (Fig. 1d and Supplementary Fig. S3). These results indicated that PMv neurons are activated by emotional stimuli, raising questions about the specific subset responsible for them. Previous studies showed that leptin receptor (LepR) neurons in the PMv stimulate the sympathetic nervous system and respond to olfactory stimulation^12^. Thus, we hypothesized that LepR neurons in the PMv promote wakefulness in response to emotional stimuli. Immunofluorescence analysis revealed that c-Fos signals mostly co-localized with LepR in the PMv after emotional stimuli (Fig. 1d, e), indicating that PMv^LepR^ neurons were indeed activated by these stimuli.

To investigate the activation of PMv^LepR^ neurons in response to emotional stimuli, we conducted fiber photometry to record calcium signals of PMv^LepR^ neurons of LepR-Cre mice (Fig. 1f). Compared to the baseline, we observed a significant increase in calcium signals immediately after all stimuli, except for control group (Fig. 1g, h), confirming that PMv^LepR^ neurons can be acutely activated by emotional stimuli. These demonstrate that the activity of PMv^LepR^ neurons is tightly correlated with emotional stimuli in both morphological and functional experiments.

To reveal the causal role between PMv^LepR^ neuron and wakefulness promotion, we employed excitatory chemogenetics in LepR-Cre mice (Fig. 1i, j). CNO administration led to a significant increase in wakefulness and a decrease in NREM and REM sleep for over a 5-h period (9:00–14:00) compared to vehicle (Fig. 1k and Supplementary Fig. S4a, b). Similarly, when injected at 19:00, CNO administration significantly increased wakefulness and decreased NREM and REM sleep over the following 5 h (Fig. 1l and Supplementary Fig. S4c, d). Furthermore, CNO administration did not affect the sleep–wake behavior of LepR-Cre mice transduced with the control virus (DIO-mCherry) during the active or inactive period (Supplementary Fig. S5). In addition, optogenetic activation of PMv^LepR^ neurons or terminals from PMv^LepR^ neurons to the medial preoptic nucleus induced an immediate transition from NREM sleep to wake and an increase of wakefulness (Supplementary Figs. S6 and S7). Our data demonstrated that activation of PMv^LepR^ neurons prolonged arousal, mimicking insomnia-like behavior.

Given that PMv^LepR^ neurons were highly responsive to emotional stimuli, we hypothesized that they drive insomnia in response to emotional stimuli. To test this, we injected hM4Di-virus in the bilateral PMv to inhibit the activity of LepR neurons, and mCherry-virus in the bilateral PMv as a control (Fig. 1m and Supplementary Fig. S8a, b). Interestingly, CNO administration did not change the total level of wakefulness, REM sleep, and NREM sleep in PMv^LepR^-hM4Di mice (Supplementary Fig. S9) and PMv^LepR^-mCherry mice (Supplementary Fig. S4) compared with vehicle. By photometry, PMv^LepR^ neurons showed minimal changes in activity during natural sleep–wake transitions (Supplementary Fig. S10). Furthermore, vehicle injection in hM4Di-expressing mice did not change the increase in the latency to NREM sleep induced by male mouse urine (Fig. 1n). However, CNO administration in hM4Di-expressing mice abolished the increase in the latency to NREM sleep induced by male mouse urine (Fig. 1n). The average increment of NREM sleep latency of PMv^LepR^-hM4Di mice with CNO and male mouse urine was dramatically decreased as compared with that in the vehicle-injected group and PMv^LepR^-mCherry mice (Fig. 1n). Consistent with these results, CNO injection induced a 67.7% decrease in wakefulness and a 4.0-fold increase in NREM sleep in PMv^LepR^-hM4Di mice after male mouse urine exposure, compared with the vehicle injection after male mouse urine exposure (Fig. 1o). Similar to male mouse urine exposure, inhibition of PMv^LepR^ neurons reduced the increase in NREM sleep latency and wakefulness, and increased the level of NREM sleep on exposure to female mouse urine (Fig. 1p, q), male rat urine (Fig. 1r, s), or TMT (Fig. 1t, u). Furthermore, in control mice only expressing mCherry, CNO administration did not change the NREM sleep latency and levels of wakefulness, NREM and REM sleep after various stimuli exposure (Supplementary Fig. S8c−j). These results revealed that PMv^LepR^ neurons are essential for emotion-induced insomnia-like behavior, rather than responsible for physiological sleep–wake regulation.

In summary, our results demonstrate that olfactory stimulation can effectively trigger positive or negative emotion (preference or avoidance) in mice. The behavior paradigms in our study may be beneficial in future research into the neuronal circuits underlying emotions. Moreover, we found that emotional stimuli prolong NREM sleep onset latency and induce wakefulness, with PMv^LepR^ neurons being activated by emotional stimuli. We further demonstrated that activation of PMv^LepR^ neurons elicited long-lasting wakefulness, mimicking insomnia-like behavior. In addition, inhibition of PMv^LepR^ neurons abolished the increase in latency to NREM sleep onset and the level of wakefulness following emotional stimuli. Our work highlights the pivotal role of PMv in emotion-induced insomnia and suggests that PMv^LepR^ neurons may be an important therapeutic target for managing emotion-induced insomnia.

### Supplementary information

Supplementary Information

## References

[1] Hall M (2004). Medicine.

[2] Cano G, Mochizuki T, Saper CB (2008). J. Neurosci..

[3] Winsky-Sommerer R (2004). J. Neurosci..

[4] Chen AX (2020). Neuron.

[5] Li SB (2020). Sci. Adv..

[6] Horio N, Murata K, Yoshikawa K, Yoshihara Y, Touhara K (2019). Nat. Commun..

[7] Hashikawa K, Hashikawa Y, Falkner A, Lin D (2016). Curr. Opin. Neurobiol..

[8] Motta, S. C. et al. *Proc. Natl. Acad. Sci. USA* **110**, 14438–14443 (2013).

[9] Stagkourakis S (2018). Nat. Neurosci..

[10] Dolensek N, Gehrlach DA, Klein AS, Gogolla N (2020). Science.

[11] Malezieux M, Klein AS, Gogolla N (2023). Annu. Rev. Neurosci..

[12] Jiang L (2020). Nat. Commun..

