# Supplementary material for: Leptin receptor neurons in the ventral premammillary nucleus modulate emotion-induced insomnia
Source: Cell Discov. 2024 Jun 4;10:59. doi: 10.1038/s41421-024-00676-x (PMC11148181; doi:10.1038/s41421-024-00676-x)
Supplement: Supplementary file 1 — Supplementary Information [file 41421_2024_676_MOESM1_ESM.pdf]

Supplementary information for

Leptin receptor neurons in the ventral premammillary nucleus modulate emotion-induced insomnia

Xiang-shan Yuan[1], Zhe Xiang[1], Jian-bo Jiang[1], Fang Yuan[2], Mu-tian Zhang[1], Kai-ying Zhang[1], Zhao-yi Chen[1], Wei-min Qu[1], Wen-sheng Li[1], Zhi-li Huang[1]

Correspondence to: yuanxiangshan1999@163.com (X.-s.Y.); wshengli88@shmu.edu.cn (W.-s.L.); huangzl@fudan.edu.cn (Z.-l.H.).

This file includes:

Materials and methods

Supplementary Reference

Supplementary Fig. S1 to S10

Materials and methods

Animals

Pathogen-free adult male LepR-Cre mice (JAX Stock No:008320) on a C57BL/6J background were obtained from Professor Sheng Wang (Hebei Medical University). Wild-type mice of C57BL/6J genetic background and wild-type Sprague Dawley (SD) rats were obtained from the Laboratory Animal Center, Chinese Academy of Sciences (Shanghai, China). A temperature-controlled facility with a 12:12 h light/dark cycle (lights on at 7:00 a.m., illumination level 100 lux) was used to house the experimental animals. Weekly cage changes were made. Water and food were available at all times. All experimental studies were performed in compliance with protocols approved by the Committee on the Ethics of Animal Experiments of Fudan University Shanghai Medical College (Permit No: 20210302-026). Every attempt was made to minimize the number of animals used and minimize their suffering during the experiment. Same-sex littermates were randomly assigned to experimental settings, and behavior assessments were conducted in a balanced fashion. All data analyses were conducted by experimenters blinded to the group and the treatment condition of the animals.

Clozapine N-oxide administration

Clozapine N-oxide (CNO) (C4759, LKT LABS, St. Paul, USA) was freshly dissolved in sterile saline before each behavior test. Mice were injected intraperitoneally (i.p.) with CNO (1 mg/kg) or vehicle (saline) at a volume of 10 mL/kg of body weight.

Urine collection

All urine was collected fresh on the day of the experiment. To collect male mouse urine, female mouse urine, or male rat urine, one animal was transferred to a fresh cage without bedding. Urine, once excreted, was pipetted from the cage, transferred into a 1.5 mL Eppendorf tube, and kept in a 4°C refrigerator until use. For emotional stimulation, 100 μL of urine or 5 μL TMT (T1608, TOKYO Chemical Industry, Japan) was spotted onto a piece of filter paper (5 cm × 5 cm). All urine- or TMT- spotted papers were presented along with another piece of filter paper spotted with saline as the control. For urine/TMT dilution, a 1/2 or 1/4 urine/TMT dilution was produced by

mixing 1 volume of undiluted urine/TMT with 1 or 3 volumes of saline.

EEG/EMG electrode-implantation surgery

Mice were fastened to a stereotaxic (RWD Life Science, Shenzhen, China) device after being continuously administered isoflurane to anesthetize them to implant electroencephalogram (EEG) and electromyography (EMG) electrodes. The implant, consisting of two stainless steel screws (1 mm in diameter) as EEG electrodes, was inserted through the skull (+1.0 mm anteroposterior; −1.5 mm mediolateral from bregma) according to the mouse brain atlas [1]. Two insulated stainless-steel wires bilaterally placed into both trapezius muscles served as EMG electrodes [2]. Dental cement was used to affix each electrode to a mini-connector and secure it to the skull. The scalp wound was closed with surgical sutures, and each mouse was kept in a warm environment until it resumed normal activity as previously described [3].

Virus injections and fiber implantations

All viruses were packaged by Brain VTA (Brain VTA Co. Ltd., Wuhan, China) and stored at −80°C before use. Mice were fixed to stereotaxic equipment after being continuously administered isoflurane through an anesthetic mask. Then 100 nL of the viral vector (AAV-hSyn-DIO-hM3Dq-mCherry, PT-0019, serotype: AAV2/9, titration: 5.14E+12 vg/mL; AAV-hSyn-DIO-hM4Di-mCherry, PT-0020, serotype: AAV2/9, titration: 5.25E+12 vg/mL; AAV-hSyn-DIO-mCherry, PT-0115, serotype: AAV2/9, titration: 5.27E+12 vg/mL; AAV-EF1α-DIO-hChR2-mCherry, PT-0002, serotype: AAV2/9, titration: 5.54E+12 vg/mL; AAV-EF1α-DIO-mCherry, PT-0013, serotype: AAV2/9, titration: 5.18E+12 vg/mL) was subsequently microinjected into the bilateral PMv (AP: −2.45mm, ML: ±0.55 mm, DV: −5.5 mm); 100 nL of AAV-EF1α-DIO-GCaMP6f (PT-0006, serotype: AAV2/9, titration: 5.00E+12 vg/mL) was injected into the left PMv of LepR-Cre mice. After the injections, the mice used for photometry recording experiments *in vivo* were unilaterally implanted with optical fibers (fiber core, 200 μm; 0.37 numerical aperture [NA], Newdoon, Hangzhou, China) above the PMv (AP: −2.45 mm, ML: − 0.55 mm, DV: −5.3 mm). For optogenetic stimulation experiments mice used *in vivo* were bilaterally implanted with optical fibers (fiber core,

200 μm; 0.37 numerical aperture [NA], Newdoon, Hangzhou, China) above the PMv (AP: - 2.45 mm, ML: ± 0.55 mm, DV: − 5.4 mm, with a 10° angle), medial preoptic nucleus (MPO) (AP: +0.9 mm, ML: ±1.3 mm, DV: −4.75 mm, with a 10° angle). The mice were kept in their cages for at least 2 weeks following the injections to ensure full recovery. Mice were sacrificed to confirm viral expression after behavioral testing.

EEG recordings and analysis

Cortical EEG and EMG signals were captured using SleepSign (Kissei Comtec, Nagano, Japan) software after being amplified, filtered, and sampled at 128 Hz (0.5–30 Hz for EEG; 20–200 Hz for EMG) as previously described [4,5]. Spectrum analysis utilizing a rapid Fourier transform in SleepSign was used to automatically grade polygraphic recordings offline (10 s epochs for chemogenetics) into the three stages of wake, rapid eye movement (REM) sleep, and non-rapid eye movement (NREM) sleep. We classified alertness as having a low-amplitude, high-frequency EEG along with a high level of EMG activity, and REM sleep as having a low-amplitude, high-frequency EEG along with no EMG activity and clearly audible theta-like (6–10 Hz) EEG activity. In the absence of motor activity, NREM sleep is represented by synchronized, high-amplitude, low-frequency (0.65–4 Hz) EEG signals.

Emotional stimuli and sleep recording

Mice were singly housed for at least 1 week prior to the introduction of stimuli. For evaluating the impact of emotional stimuli on latency to sleep onset and general sleep architecture, adult male mice (10–14 weeks old, wild-type mice and LepR-Cre mice) with EEG/EMG implants (for sleep pattern monitoring) were exposed to different urine/TMT with a narrow window on top for EEG/EMG cable sliding. Moreover, we determined the value of the latency increment (Δ Latency) by calculating $(L–L_0)/L_0$, where L is the increment of latency to NREM sleep of each mouse, and L_0 is the median increment of latency to NREM sleep of each group. For emotional stimuli, a small piece of filter paper with 100 μL of saline/male mouse urine/female mouse urine/male rat urine or 5 μL of TMT was introduced into the home cage of the animal. We introduced each stimulus at 10:00 a.m. when mice had a strong urge to sleep.

Fiber photometry

Mice were housed in their home cages for recovery for at least 2 weeks to achieve sufficient viral expression before they were connected to the EEG/EMG recording cables and fiber photometry recording patch cord. The emotional stimuli were recorded with the mice housed individually with 1-week habituation to the fiber patch cord. The GCaMP6f signal was sampled for 4 min each session, with 2 min as the baseline and a 2 min exposure to the control (saline) and emotional stimuli (male mouse urine, female mouse urine, male rat urine, or TMT). Stimuli were removed immediately after the 2-minute exposure. The introduction of salient stimuli to each mouse was randomized. For EEG/EMG recording, fiber-photometry experiments were performed in both light and dark periods as previously described [6,7]. Photometry data were exported to MATLAB Mat files from Spike2 for further analysis. We derived the value of the photometry signal ($\Delta F/F$) by calculating $(F–F_0)/F_0$, where F_0 is the median fluorescent signal. The averaged $\Delta F/F$ was calculated during all sleep–wake state times. For analyzing state transitions, we determined each state transition and aligned $\Delta F/F$ in a ± 50-s window around each calculated point. The average peak of the $\Delta F/F$ was selected and compared for different sleep stages.

Optogenetic stimulation *in vivo*

For *in vivo* optogenetic stimulation, light-pulse trains were generated via a laser stimulator (BL473T3-100FC, Shanghai laser Century, China) and output through an isolator (ss-102J, Nihon Kohden, Japan). To reduce torque, a rotating optical junction (FRJFC-FC, Doric Lenses, Canada) was fastened to the optical fiber's outside end. For acute photo-stimulation, each stimulation epoch was applied 20 s after identifying a stable NREM sleep event by real-time online EEG/EMG analysis. The light period, when mice are not active, was set and used to provide light-pulse trains (10 ms in length each). From 9:00 to10:00, scheduled light-pulse trains (10-ms pulses at 20 Hz, with 20-s on/20-s off for 1h) were employed for chronic photo-stimulation. Chronic photo-stimulation programmed light-pulse trains (continuous pulses, with 40 s on/ 20 s off for 1 h) were utilized from 21:00 to 22:00 to optogenetically block PMv LepR neurons.

EEG/EMG recordings during the same period on the previous day were served as a baseline control. Before each experiment, the power of the light was measured using a power meter (PM10, Coherent) that was set to emit 5 mW/mm^2 of light from the tip of the optical fiber cannula [8].

Immunohistochemistry

Immunohistochemistry was performed as described previously [3,9]. At 1.5 h following CNO administration or emotional stimulation, mice were administered a deep anesthetic and transcardially perfused with 10 mL saline, followed by 100 mL of 4% paraformaldehyde in 0.1 M phosphate buffer (PB, pH 7.2). The brains were removed, postfixed for 4–6 h at 4°C, and then cryoprotected in 30% sucrose in 0.1 M PB at 4°C until they sank. Coronal slices (30 μm) from each brain were obtained in three series using a microtome (CM1950, Leica, Germany) and collected in 0.01 M phosphate-buffered saline (pH 7.2). For c-Fos and LepR dual-staining, incubation with a primary antibody was performed at 4°C for 48 h (rabbit anti-c-Fos, 1:5,000; ab190289, Abcam, UK; mouse anti-c-Fos, 1:5,000; ab208942, Abcam, UK; rabbit anti-LepR, 1:1000; ab5593, Abcam, UK). Following a PBS wash, sections were treated with a secondary antibody (Alexa Fluor 488 donkey anti-rabbit IgG, 1:1000; Alexa Fluor 488 donkey anti-mouse IgG, 1:500; Alexa Fluor 594 donkey anti-rabbit IgG, 1:500, all from Jackson ImmunoResearch, West Grove, USA) for 2 h at room temperature. The sections were then incubated in PBS containing DAPI (1:3000, D9542, Sigma-Aldrich, Sigma-Aldrich, USA) for 10 min. Finally, sections were washed in PBS and coverslipped with Fluoromout-G™ (0100-01, Southern Biotech, Birmingham, USA). Fluorescence images were collected using a laser confocal microscope (SP8, Leica, Germany) or Olympus VS120 microscope (Japan).

Electron microscopy

Under deep anesthesia, LepR-Cre mice injected with AAV-DIO-ChR2-mCherry, were briefly perfused transcardially with 6–8 mL saline followed by 100 mL ice cold fixative containing 4% paraformaldehyde, 0.5% glutaraldehyde, and 15% saturated picric acid in 0.1 M PB (pH 7.4). Immediately after perfusion-fixation, brains were removed and

postfixed in 4% paraformaldehyde for an additional 2 h at 4°C.

Brain samples containing the PMv and MPO were cut into coronal sections (50 μm thick) with a vibratome (VT1000S, Leica) and collected in 0.05 M PB for mCherry staining. The basic immunohistochemical protocols were the same as those described above. Briefly, the sections were placed in 0.05 M PB (pH 7.4) containing 25% (w/v) sucrose and 10% (v/v) glycerol for 1 h and then freeze-thawed with liquid nitrogen to enhance antibody penetration [3]. The sections were then incubated in rabbit anti-mCherry antibody (1:3000, 632496, Takara, Japan) in 0.05 M PB containing 5% normal donkey serum for 24 h at 4°C. Next, the sections were incubated with donkey anti-rabbit biotinylated IgG (1:1000, Jackson ImmunoResearch) for 3 h at RT, followed by incubation in ABC complex for 3 h at RT. Some sections containing the PMv or MPO were incubated in DAB peroxidase substrate (SK-4100, Vector Laboratories, USA) to observe the expression of mCherry in LepR neurons of the PMv or the terminals in the MPO. The labeled sections were osmicated with 2% OsO4, dehydrated in a graded series of ethanol, and embedded flat in Epon 12 (Ted Pella, Redding, USA) using embedding capsules (TAAB, Berks, UK). The sections embedded in Epon were observed under a light microscope and areas of the MPO were sampled. Ultrathin sections were cut at a thickness of 70 nm using an ultramicrotome, stained with uranyl acetate and lead citrate, and then examined in a CM-120 transmission electron microscope (Philips, Netherlands).

***In vitro* electrophysiology**

In vitro electrophysiological experiments were performed as described below. After virus injections in LepR-Cre mice for 3–4 weeks, mice were anesthetized and perfused transcardially with ice-cold modified aCSF saturated with 95% O_2 and 5% CO_2 containing the following (in mM): 215 sucrose, 26 $NaHCO_3$, 10 glucose, 3 $MgSO_4$, 2.5 KCl, 1.25 NaH_2PO_4, 0.6 Na-pyruvate, 0.4 ascorbic acid, and 0.1 $CaCl_2$. Then, the brains were rapidly removed into an ice-water mixture, and acute coronal slices (300 μm) containing the PMv were cut on a vibratome (VT1200s, Leica, Germany) in an ice-cold mixture of modified aCSF with 95% O_2 and 5% CO_2. Next, the slices were transferred

to a holding chamber containing normal recording aCSF (in mM: 125 NaCl, 26 $NaHCO_3$, 25 glucose, 2.5 KCl, 2 $CaCl_2$, 1.25 NaH_2PO_4, and 1.0 $MgSO_4$). Slices were incubated for 30 min at 36.5°C and equilibrated for at least 30 min at room temperature (22°C) before recordings were obtained.

Slices were transferred to the submersion recording chamber and continuously perfused with oxygenated aCSF at 32°C at a rate of 2 mL/min while the recordings were taken. Neurons in the PMv were identified under visual direction using infrared-differential interference contrast video microscopy with a water immersion objective lens (40×, BX51WI, Olympus). Images were captured with an infrared-sensitive CCD camera (PHOTOMETRICS, Iris 9). In LepR-Cre mice, LepR neurons were identified by mCherry expression. Recordings were performed in regions with bright mCherry fluorescence. Recording pipettes (5–8 MΩ) were filled with an internal solution containing the following (in mM): 105 potassium gluconate, 30 KCl, 10 phosphocreatine, 4 ATP-Mg, 0.3 EGTA, 0.3 GTP-Na, and 10 HEPES (pH 7.3, 285–300 mOsm). Recordings were conducted in the whole-cell or cell attached configuration using a Multiclamp 700B amplifier (Axon Instruments, USA). Signals were filtered at 2 kHz and digitized at 10 kHz with a DigiData 1440 A (Axon Instruments). Data were acquired and analyzed with pClamp10.3 software (Axon Instruments). In the voltage-clamp mode, cells were held at −70 mV. Cells with Ra changes over 20% were discarded.

Conditioned place preference test

Mice were singly housed for at least 1 week prior to the conditioned place preference (CPP) test. The CPP test was conducted in a specially constructed behavioral arena (45 × 45 × 40 cm black acrylic glass) with an overhead lamp pointed toward the field's center and 50 lux of illumination on the arena's floor. Before the day of CPP testing, all mice underwent a single 5-min acclimatization session. The behavioral arena was divided into nine equally sized subregions using behavioral analysis software (Tracking Master, Fanbi Intelligent Technology Co., Ltd, Shanghai, China). The subregion in the upper left corner was assigned as area A, and the lower right corner was assigned as

area B. A piece of small filter paper with 100 μL of male mouse urine/female mouse urine/male rat urine or 5 μL of TMT was introduced into area A, and a piece of small filter paper with 100 μL saline was introduced into area B. Mice were placed into the arena for 10 min, during which time a video was recorded to analyze the time spent in either area A or area B. Each animal was tested once regarding its emotional stimulus preference.

Cell counting

Brain areas were determined according to the Adult Mouse Coronal Atlas [1]. Brain section images were taken using an Olympus VS120 microscope (Japan) and a Leica confocal microscope (SP8). Quantification of c-Fos staining was performed by number/section/bilateral side. The c-Fos^+ cell number of each nucleus was manually counted with Image Pro Plus software (Media Cybernetics Inc., USA) from 1/3 of the brain sections of each mouse.

Statistical analysis

Sample sizes were determined based on previous studies using chemogenetics, and fiber photometry to investigate neural circuits underlying sleep–wake state regulation and other behaviors [7,10]. Data are expressed as the mean ± standard error of the mean (sem). Paired and unpaired *t* tests were used for comparisons between two groups. One-way analysis of variance (ANOVA) was used to compare more than two groups. Two-way ANOVA or ANOVA for factorial design were used to perform multiple group comparisons. All statistical tests were two-tailed. Statistical significance was considered when $p < 0.05$. All data were analyzed using SPSS 20 software (IBM, Armonk, USA); $^{*}p < 0.05$, $^{**}p < 0.01$.

Reference:

1 Paxinos George & Franklin, K. B. J. *The mouse brain in stereotaxic coordinates*. 2nd edition edn, (Academic Press, 2001).

2 Luo, Y. J. *et al. Nat Commun* **9**, 1576 (2018).

3 Yuan, X. S. *et al. eLife* **6**, e29055 (2017).

4 Huang, Z. L. *et al. Proc. Natl. Acad. Sci. U. S. A.* **103**, 4687-4692 (2006).

5 Huang, Z. L. *et al. Proc. Natl. Acad. Sci. U. S. A.* **98**, 9965-9970 (2001).

6 Li, Y. D. *et al. Mol. Psychiatry* **26**, 2912-2928 (2020).

7 Dong, H. *et al. Curr. Biol.* **32**, 600-613 (2022).

8 Chen, Z. K. *et al. Cell discovery* **8**, 115 (2022).

9 Guo, H. *et al. Neurosci. Bull.* **36**, 585-597 (2020).

10 Eban-Rothschild, A., Rothschild, G., Giardino, W. J., Jones, J. R. & de Lecea, L. *Nat. Neurosci.* **19**, 1356-1366 (2016).

Supplementary Figure and legend

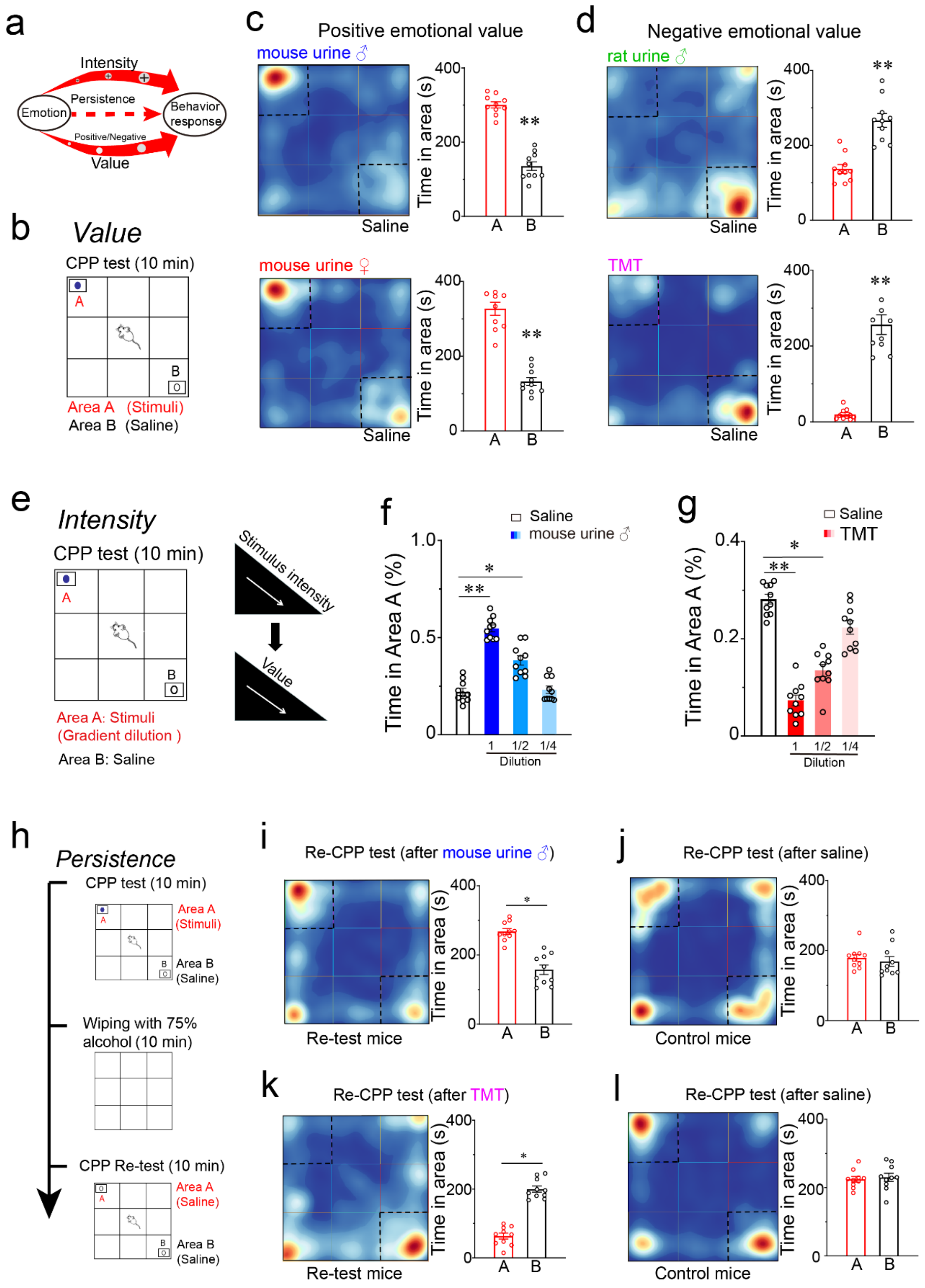

Supplementary Fig. S1 Olfactory stimulation induced core features of emotion states.

a Schematic of a multidimensional model for core features of emotion states. **b** Schematic plot showing experiment protocol: a piece of small filter paper (5 cm×5 cm) with 100 μL male mouse urine/female mouse urine/male rat urine or 5 μL TMT were respectively placed into area A, and a piece of small filter paper (5 cm×5 cm) with 100 μL saline

were introduced into area B. Mice were brought to the open field arena and explored freely for 10 min. **c** Representative conditioned place preference tests heatmaps (left panel) and column (right panel) showed the length of time which mice spent in area A with male/female mouse urine as a positive emotional value for mice, and B with saline (n = 10, independent-samples Student's *t* tests). **d** Representative conditioned place preference tests heatmaps (left panel) and column (right panel) showed the length of time which mice spent in area A with male rat urine/TMT as a negative emotional value for mice, and B with saline (n = 10, independent-samples Student's *t* tests). **e** Schematic showed that experiment protocol evaluated the effect of different intensity of stimuli on emotional value. **f** Percentage time spent in area A when animals sniffed different dilutions of male mouse urine (n = 10, one-way ANOVA). **g** Percentage time spent in area A when animals sniffed different dilutions of TMT (n = 10, one-way ANOVA). **h** Schematic showed that experiment protocol evaluated persistence of stimuli on emotional value. **i** Representative conditioned place preference tests heatmaps (left panel) and column (right panel) showed the length of time which Re-test mice (after male mouse urine) spent in area A with saline and area B with saline (n = 10, independent-samples Student's *t* tests). **j** Representative conditioned place preference tests heatmaps (left panel) and column (right panel) showed the length of time which control mice (no-training for male mouse urine) spent in area A with saline and area B with saline (n = 10, independent-samples Student's *t* tests). **k** Representative conditioned place preference tests heatmaps (left panel) and column (right panel) showed the length of time which Re test mice (after TMT) spent in area A with saline and area B with saline (n = 10, independent-samples Student's *t* tests). **l** Representative conditioned place preference tests heatmaps (left panel) and column (right panel) showed the length of time which control mice (no-training for TMT) spent in area A with saline and area B with saline (n = 10, independent-samples Student's *t* tests). Data represent mean ± sem, *$p < 0.05$, **$p < 0.01$.

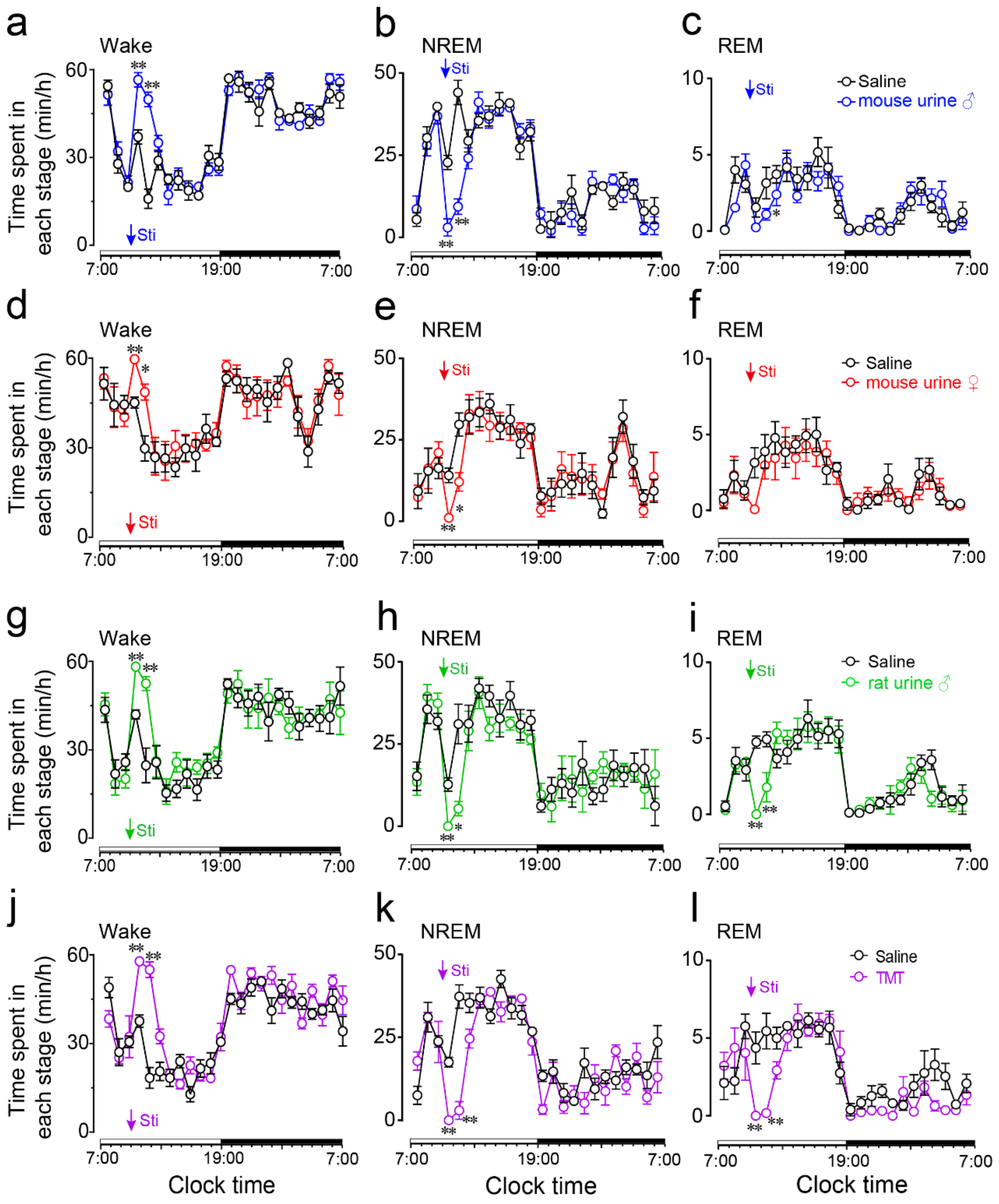

Supplementary Fig. S2 Emotional stimuli induced wakefulness.

a, d, g, j Time course of wakefulness following male mouse urine (**a**, open blue circle), female mouse urine (**d**, open red circle), male rat urine (**g**, open green circle), TMT (**j**, open magenta circle) and saline (open black circle) stimuli (n = 6, two-way repeated measures ANOVA). **b, e, h, k** Time course of NREM sleep following male mouse urine (**b**, open blue circle), female mouse urine (**e**, open red circle), male rat urine (**h**, open green circle), TMT (**k**, open magenta circle) and saline (open black circle) stimuli (n = 6, two-way repeated measures ANOVA). **c, f, i, l** Time course of REM sleep following male mouse urine (**c**, open blue circle), female mouse urine (**f**, open red circle), male rat urine (**i**, open green circle), TMT (**l**, open magenta circle) and saline (open black circle) stimuli (n = 6, two-way repeated measures ANOVA). Data represent mean ± sem, *$p < 0.05$, **$p < 0.01$.

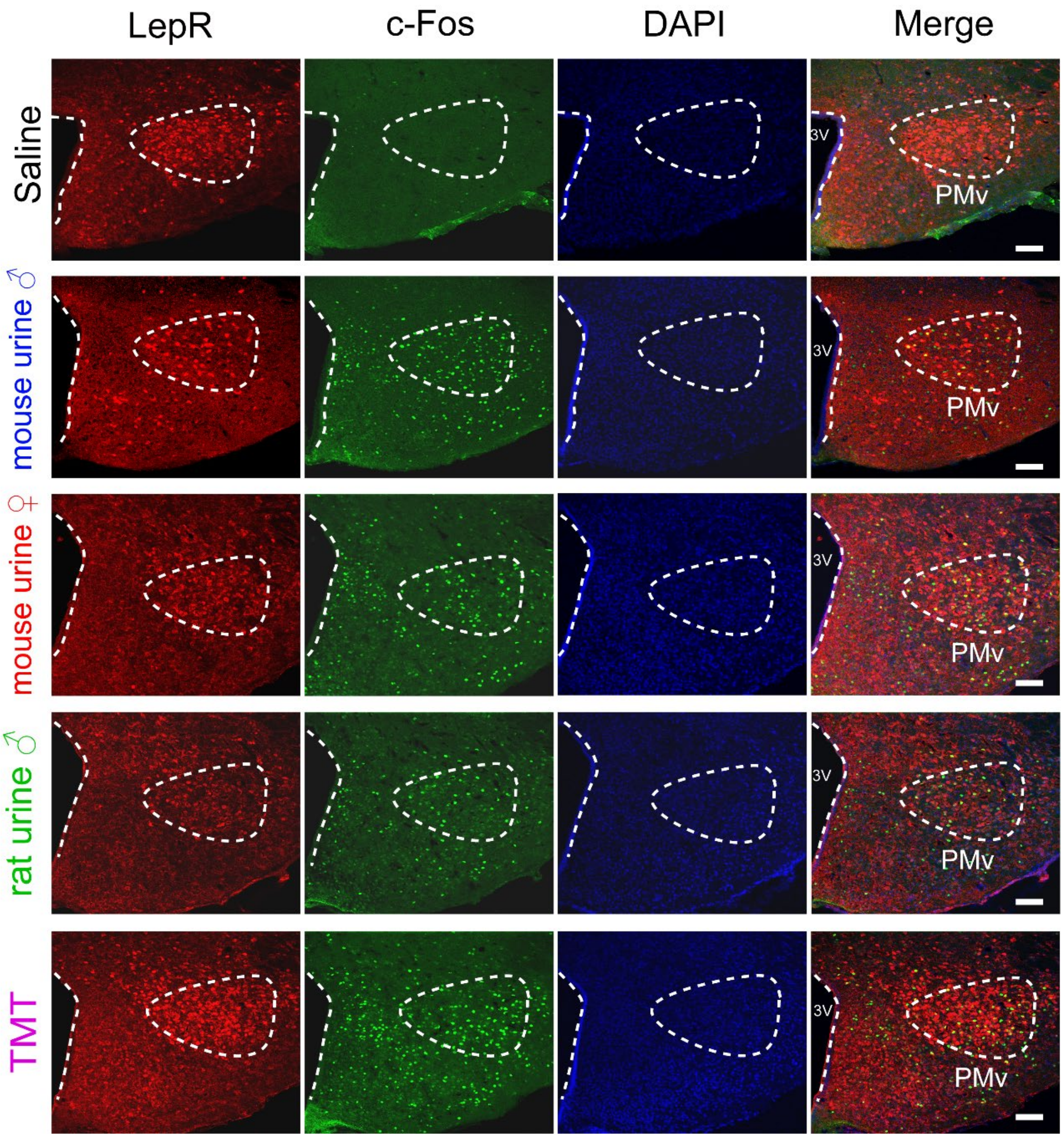

Supplementary Fig. S3 Colocalization of c-Fos and LepR signals in the PMv following different odor stimuli.

Representative images showing colocalization of c-Fos (green) and LepR (red) signals in the PMv for a saline and an experimental animal exposed to male mouse urine/female mouse urine/male rat urine/TMT, respectively. Scale bar, 200 μm.

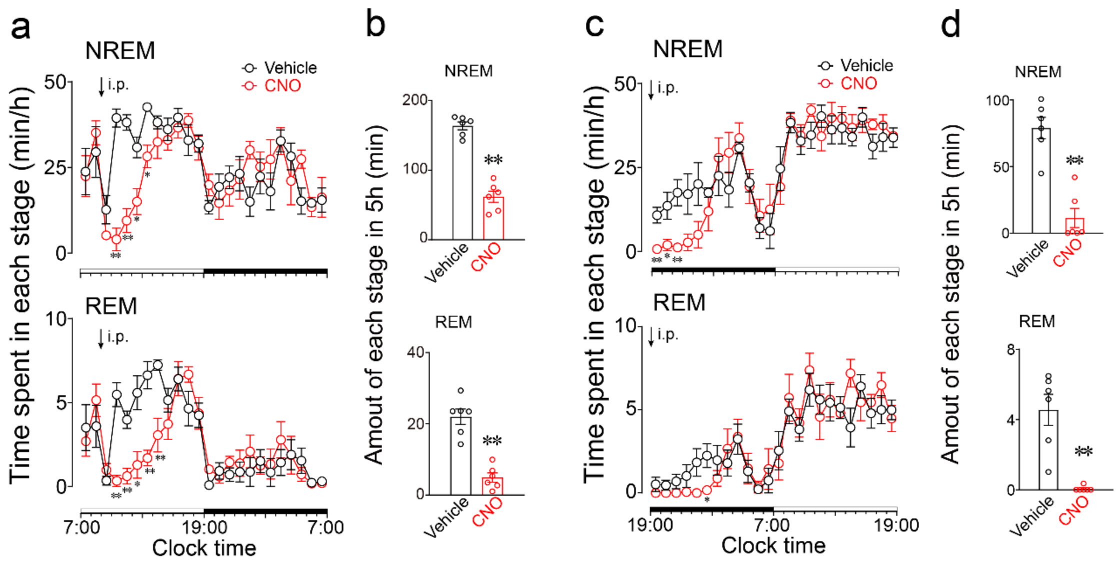

Supplementary Fig. S4 Chemogenetic activation of PMv^{LepR} neurons decreased NREM, REM sleep.

a Time course of NREM (upper panel) and REM (lower panel) sleep following vehicle (open black circle) or CNO (closed red circle) administration at 9:00 to PMv^{LepR}-hM3Dq-mCherry mice (n = 6, two-way repeated measures ANOVA). **b** Total time spent in each stage for 5 h (9:00 - 14:00) after vehicle or CNO administration (n = 6, paired *t* test). **c** Time course of NREM (upper panel) and REM (lower panel) sleep following vehicle (open black circle) or CNO (open red circle) administration at 19:00 to PMv^{LepR}-hM3Dq-mCherry mice (n = 6, two-way repeated measures ANOVA). **d** Total time spent in each stage for 5 h (19:00 - 24:00) after vehicle or CNO injection (n = 6, paired *t* test). Data represent mean ± sem, $*p < 0.05$, $**p < 0.01$.

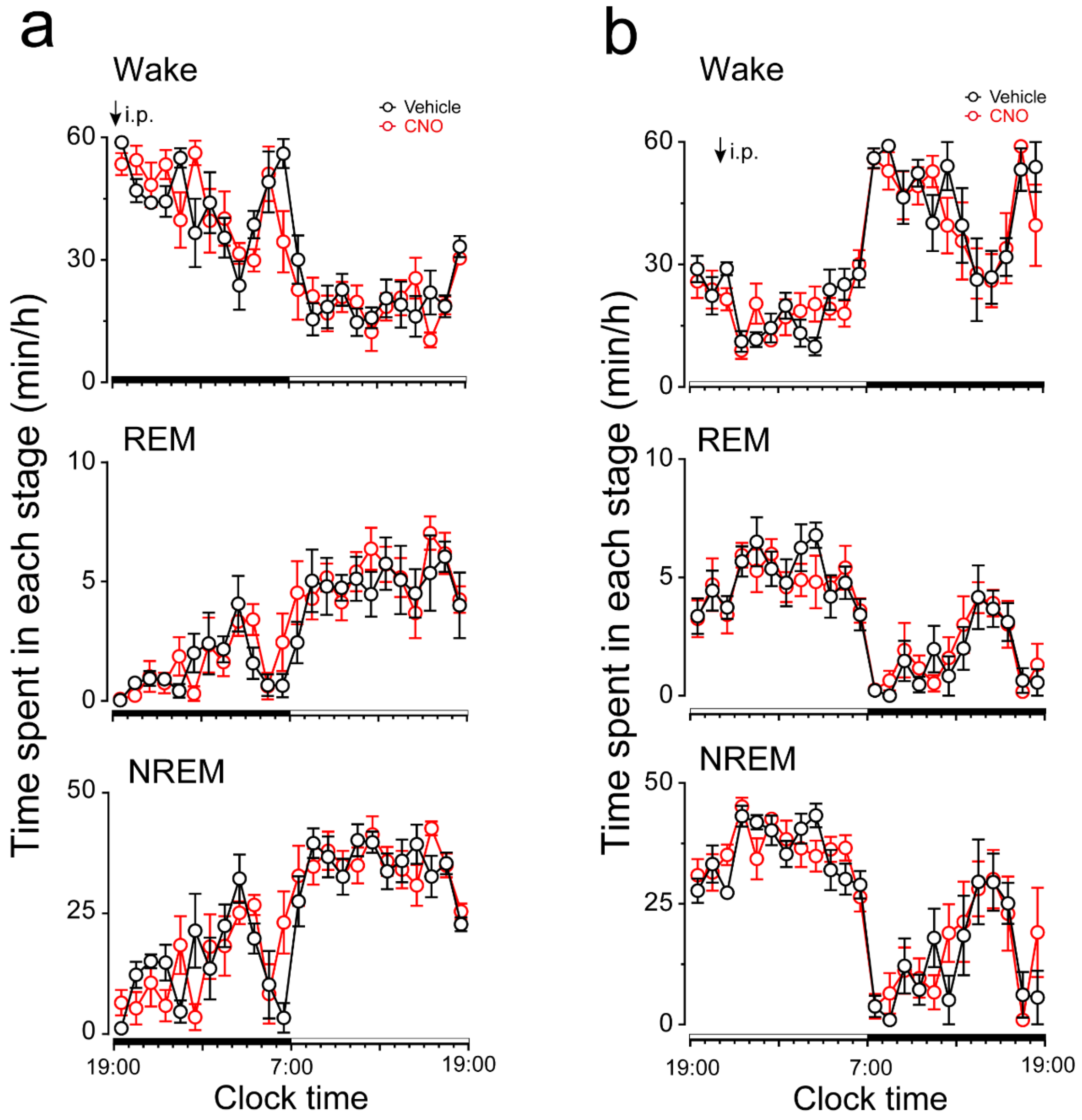

Supplementary Fig. S5 CNO administration did not alter sleep-wake stage of PMv^{LepR} neurons only expressing mCherry.

a Time course of wakefulness, NREM and REM sleep following vehicle (open black circle) or CNO (closed red circle) administration at 19:00 to PMv^{LepR}-mCherry mice (n = 6, two-way repeated measures ANOVA). **b** Time course of wakefulness, NREM and REM sleep following vehicle (open black circle) or CNO (closed red circle) administration at 9:00 to PMv^{LepR}-mCherry mice (n = 6, two-way repeated measures ANOVA). Data represent mean ± sem.

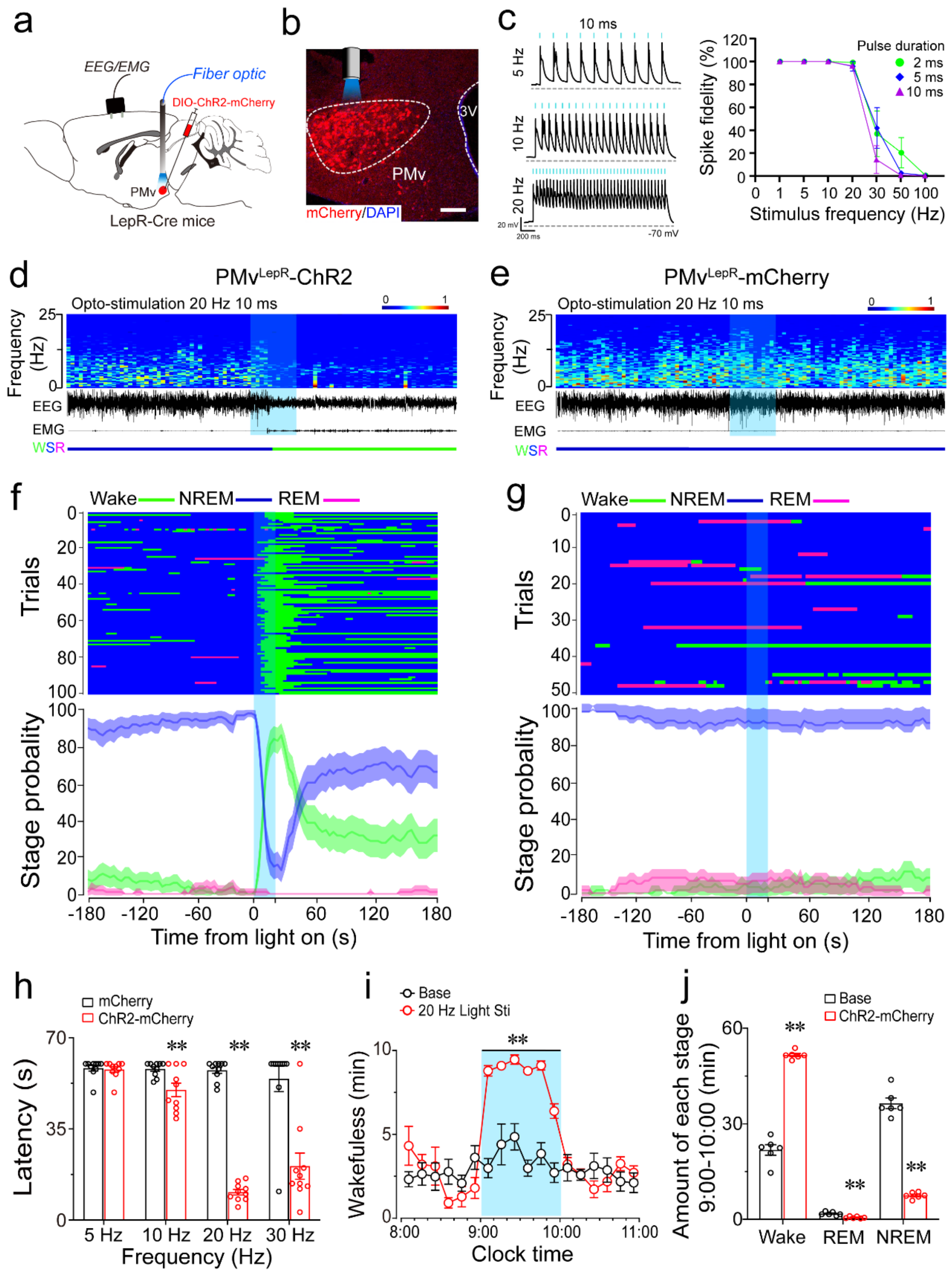

Supplementary Fig. S6 Optogenetic activation of PMv LepR neurons promotes arousal

a Sagittal diagram for in vivo optical stimulation and EEG/EMG recording. **b** Representative image showing the expression of ChR2-mCherry in the PMv of LepR-Cre mouse. The track showing the placement of optic fiber above the PMv. Scale bar, 200 μm. **c** Quantification of spike fidelity in relation to photo-stimulation frequency and pulse duration (n=10 neurons from 4 mice). **d, e** Representative EEG/EMG traces, heat map of EEG power spectra showed that acute photo-stimulation (20 Hz/10 ms) applied during NREM sleep induced an immediate transition to wake in a ChR2-mCherry mouse (**d**), but not in mCherry control mouse (**e**). **f, g** Sleep stages after PMv LepR neurons blue-light stimulation in ChR2-mCherry mice (**f**) or mCherry control mice (**g**). **h** Latencies of transitions from NREM

sleep to wakefulness after photo-stimulation at different frequencies (10 trials for each frequency from 6 mice). **i** Time course of wakefulness during semi-chronic optogenetic stimulation (20 Hz/10ms, 20 s on/20 s off) to PMv^{LepR} neurons (n = 6, two-way repeated measures ANOVA). The bule column indicates the photo-stimulation period of the stimulation group. **j** Total amounts of wakefulness, NREM and REM sleep in control group and optogenetic stimulation group (n = 6, paired *t* test). Data represent mean ± sem. *p < 0.05, **p < 0.01.

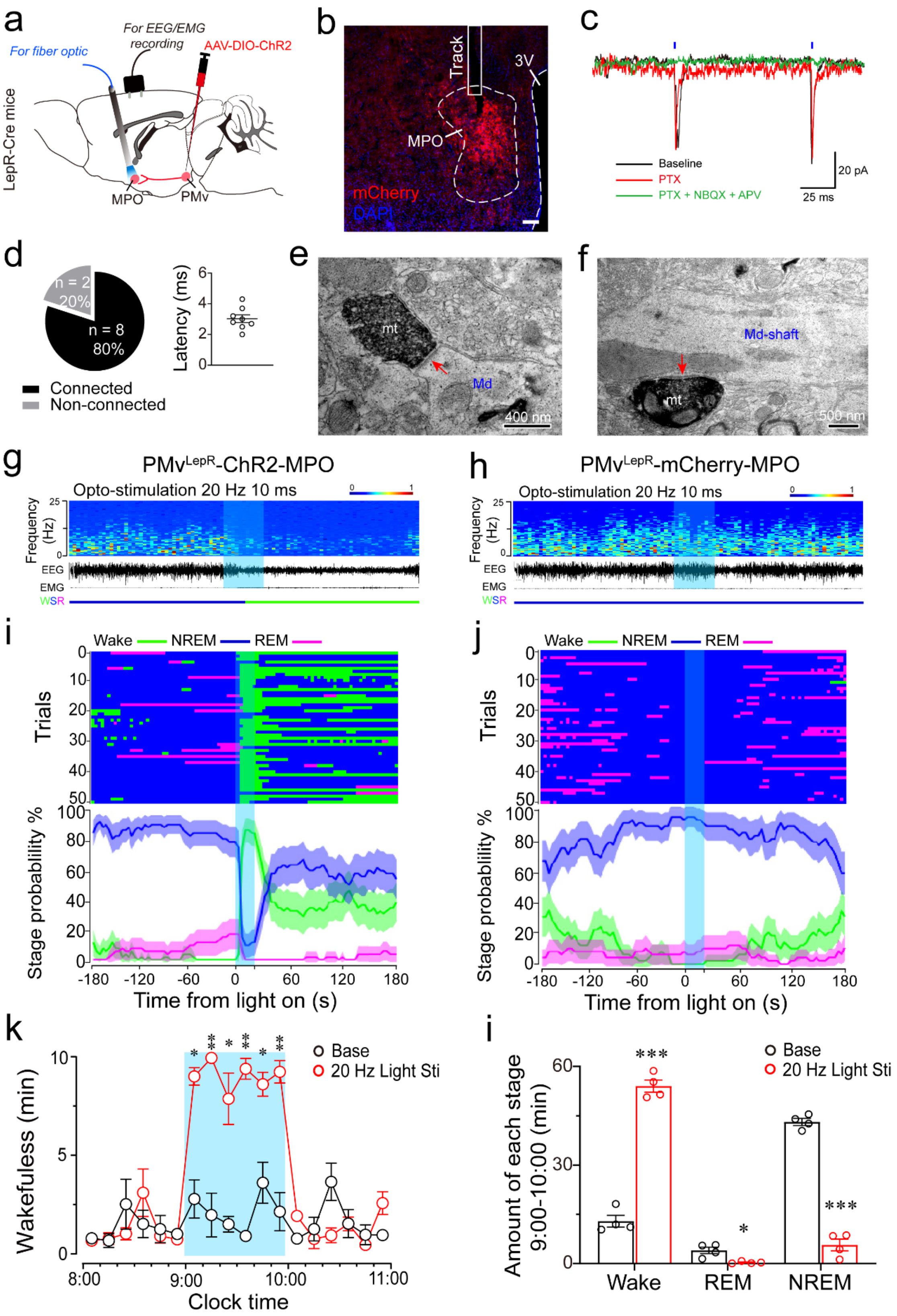

Supplementary Fig. S7 Photo-stimulation of PMv-MPO projections promotes wakefulness

a Sagittal diagram for in vivo optogenetic stimulation of the PMv^{LepR}-MPO pathway in LepR-Cre mice. **b** Representative image for mCherry showing ChR2-mCherry-positive terminals in the MPO, and the track showing the optic fibers targeting above the PMv. Scale bars: 200 μm. **c** Representative traces of excitatory postsynaptic

currents evoked on baseline (black), in picrotoxin (PTX) (orange), and in PTX + NBQX (a highly selective AMPA receptor antagonist) + DL-2-amino-5-phosphonovaleric acid (APV) (green) is shown. Scale bars, 20 pA, 25 ms. **d** Eighty percent of MPO neurons showed connections with PMv LepR neurons (n = 10 cells from 3 mice). Latency of light-evoked excitatory postsynaptic currents in MPO neurons. **e, f** Electron microscopy image showed that mCherry-immunoreactive terminal (mt) that formed an asymmetric synapse (arrow) with MPO-positive dendrite (Md). Scale bar: 400 nm (**e**), Scale bar: 500 nm (**f**). **g, h** Representative EEG/EMG traces, heat map of EEG power spectra show that photo-stimulation (20 Hz/10 ms) during NREM sleep induced a transition to wake in a PMv^{LepR}-ChR2-MPO mouse (**g**), but not in PMv^{LepR}-mCherry-MPO mouse (**h**). **i, j** Sleep stages after MPO blue-light stimulation in ChR2-mCherry mice (**i**) or mCherry control mice (**j**). **k** Time course of wakefulness during semi-chronic optogenetic experiment (20 Hz/10ms, 20 s on/20 s off). The blue column indicates the photo-stimulation period of the stimulation group (n = 4, two-way repeated measures ANOVA). **l** Total amounts of each stage in control and photo-stimulation groups (n = 4, paired *t* test). Data represent mean ± sem. *$p < 0.05$, **$p < 0.01$.

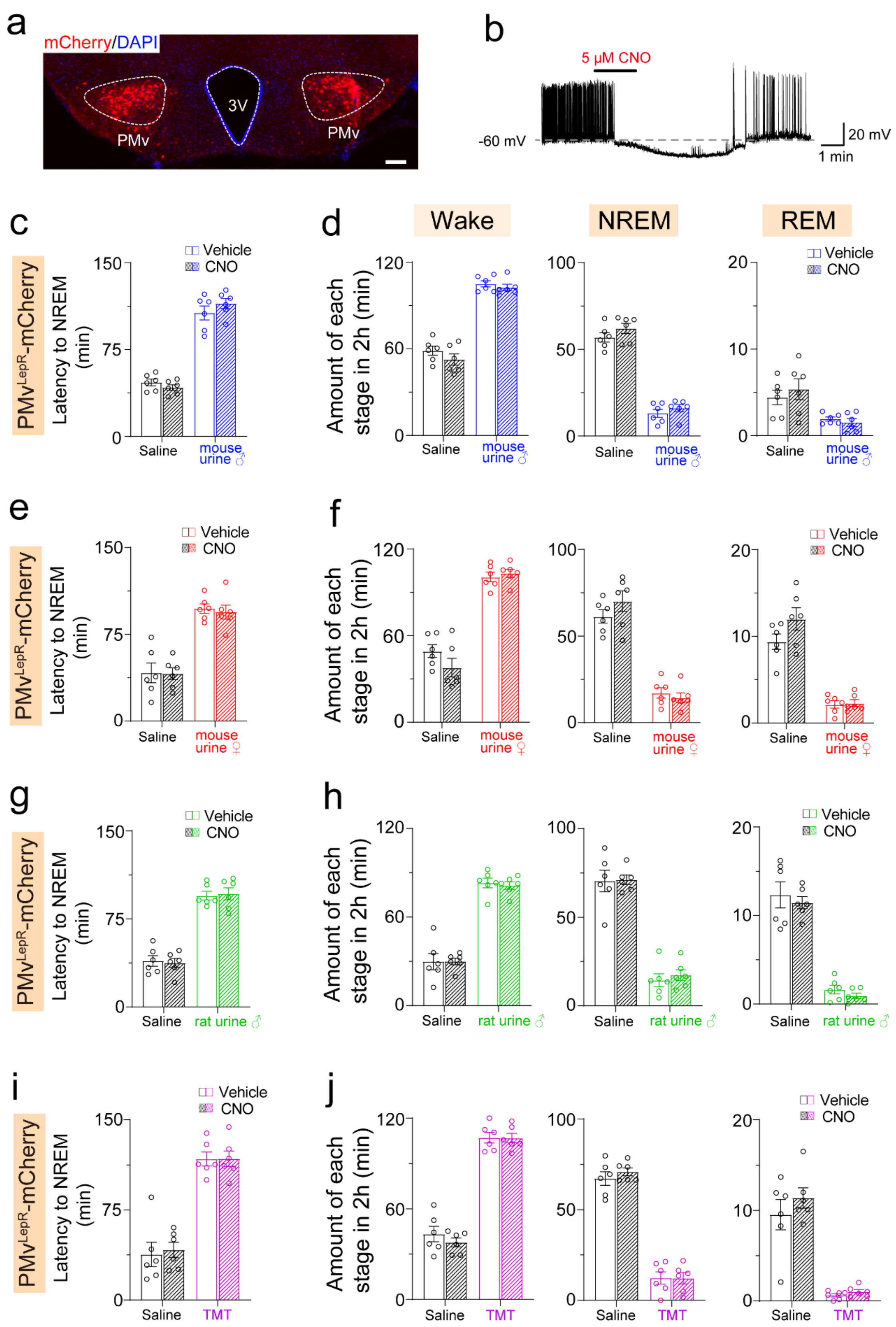

Supplementary Fig. S8 Effects of CNO administration on PMv^{LepR}-mCherry group.

a Representative image showing mCherry (red) expression in the PMv of PMv^{LepR}-hM4Di mouse. Scale bar, 200 μm. **b** Bath application of CNO at 5 μM decreased firings in a hM4Di-mCherry neuron in the PMv of LepR-Cre mouse. **c, e, g, i** The latency of NREM sleep following vehicle or CNO administration in PMv^{LepR}-mCherry mice

after exposure to male mouse urine (**c**), female mouse urine (**e**), male rat urine (**g**), TMT (**i**) or saline (n = 6, ANOVA for factorial design). **d, f, h, j** Total amount of wakefulness, NREM and REM sleep during the 2 h (10:00-12:00) following vehicle or CNO injections in PMv^{LepR}-mCherry mice after exposure to saline or male mouse urine (**d**), female mouse urine (**f**), male rat urine (**h**), TMT (**j**) (n = 6, paired *t* test). Data represent mean ± sem.

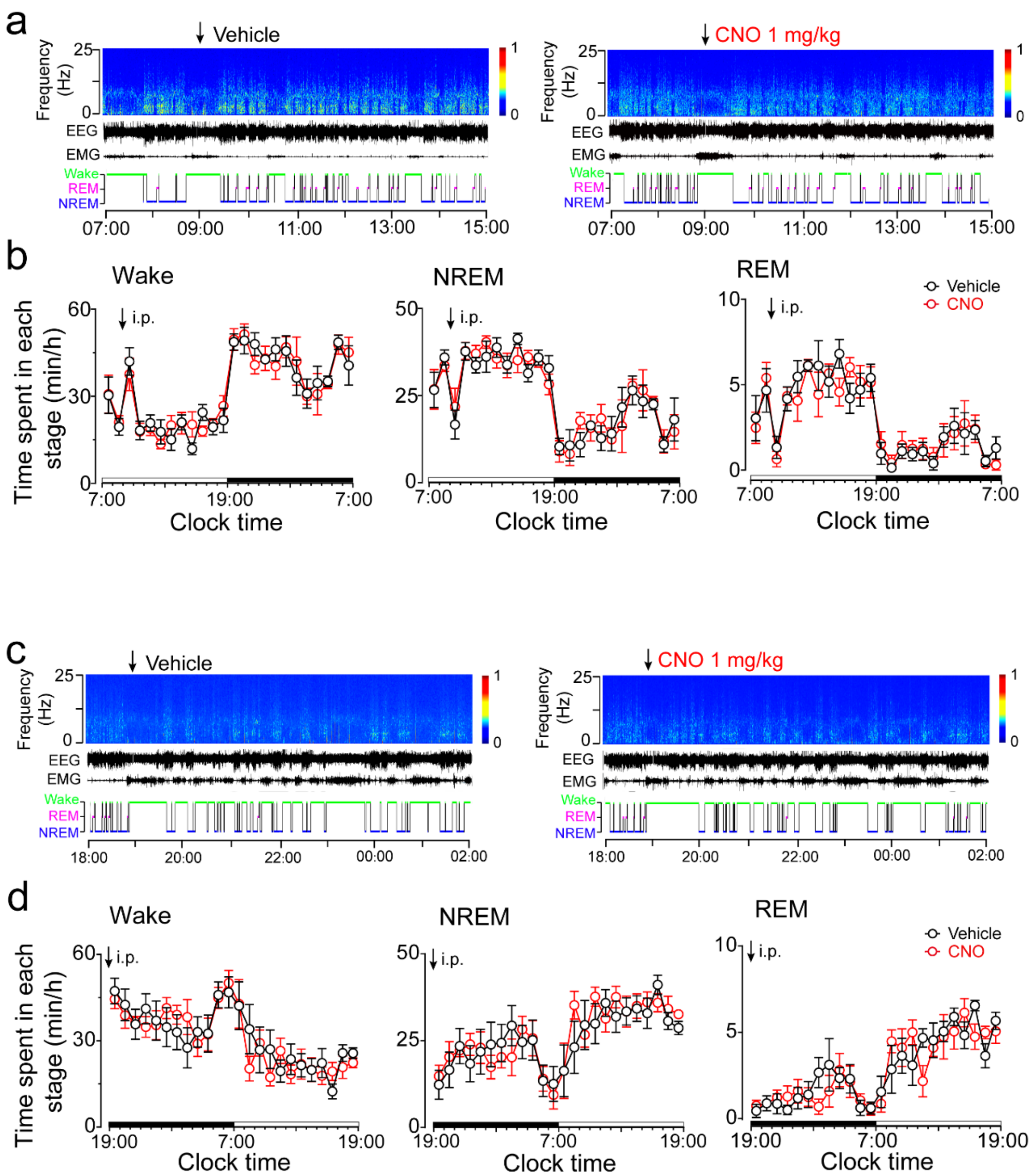

Supplementary Fig. S9 Chemogenetic inhibition of PMv^{LepR} neurons did not affect the sleep/wake pattern.

a Examples of relative EEG power, EEG/EMG traces, and hypnograms over 6 h following vehicle or CNO injection at 9:00. **b** Time course changes in wakefulness, REM and NREM sleep after administration of vehicle (black circle) or CNO (red circle) to PMv^{LepR}-hM4Di-mCherry mice (n = 6, two-way repeated measures ANOVA). **c** Examples of relative EEG power, EEG/EMG traces, and hypnograms over 6 h following vehicle or CNO injection at 19:00. **b** Time course changes in wakefulness, REM and NREM sleep after administration of vehicle (black circle) or CNO (red circle) to PMv^{LepR}-hM4Di-mCherry mice (n = 6, two-way repeated measures ANOVA). Data represent mean ± sem.

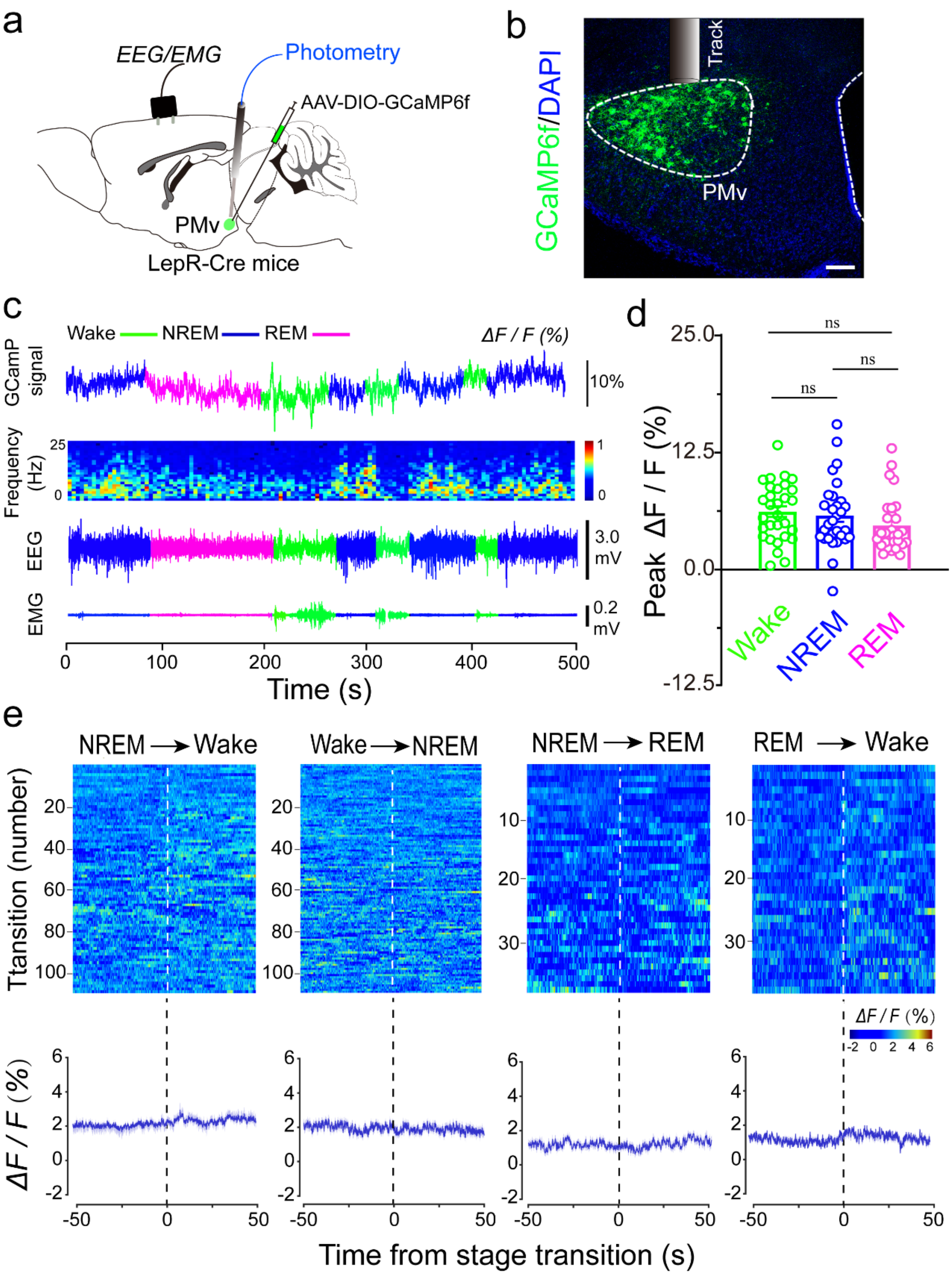

Supplementary Fig. S10 Population activity of PMv LepR neurons across sleep-wake states

a Schematic of in vivo fiber-photometry recordings. **b** A representative image showing unilateral GCaMP6f (green) expression in the PMv of LepR-Cre mouse. Blue represents DAPI staining. Scale bar, 200 µm. The cannula trace showing the optic fibers targeting above the PMv. **c** Representative fluorescence traces, relative EEG power, and EEG/EMG traces across spontaneous sleep-wake states. **d** ΔF/F peaks during wakefulness, NREM sleep, and REM sleep. The fluorescent peak values were normalized by the mean ΔF/F peaks during NREM sleep (n = 3 mice, ten sessions per mouse, one-way ANOVA, followed by Tukey's multiple tests). **e** Fluorescence signals aligned to

arousal-state transitions. Upper panel: Individual transitions with color-coded fluorescent intensities. Lower panel: Average calcium transients from all the transitions. Data represent mean ± sem.
